# *Arthrostoma leucurus* sp. n. (Nematoda: Ancylostomatidae), A New Hookworm Species Isolated from Asian Badger in China

**DOI:** 10.1007/s11686-022-00587-5

**Published:** 2022-07-23

**Authors:** Gang Liu, Shiyi Wang, Weihua Liang, Sándor Hornok, Shanshan Zhao, Wenbo Tan, Zhiqiang Liu, Xinli Gu, Yuanzhi Wang

**Affiliations:** 1 School of Medicine, Shihezi University, Shihezi, 832002 Xinjiang Uygur Autonomous Region People’s Republic of China; 2 College of Animal Science and Technology, Shihezi University, Shihezi, 832002 Xinjiang Uygur Autonomous Region People’s Republic of China; 3 NHC Key Laboratory of Prevention and Treatment of Central Asia High Incidence Diseases, First Affiliated Hospital, School of Medicine, Shihezi University, Shihezi City, Xinjiang Uygur Autonomous Region People’s Republic of China; 4 Department of Parasitology and Zoology, University of Veterinary Medicine, Budapest, 1078 Hungary; 5 Institute of Veterinary Medicine, Xinjiang Academy of Animal Science, Ürümqi, Xinjiang Uygur Autonomous Region People’s Republic of China

**Keywords:** Hookworm, Ancylostomatoidea, Morphological examination, *Cox*1

## Abstract

**Purpose:**

To date, ten validated *Arthrostoma* species were reported. Here, a new hookworm species was found from Asian badger (*Meles leucurus*).

**Methods:**

Nineteen hookworms (9 males and 10 females) were collected from the small intestine of two Asian badgers in Xinjiang Uygur Autonomous Region, northwestern China. The hookworms were morphologically examined according to key taxonomic characters, such as anterior extremity direction, structures of oral opening (cutting plates or teeth), vulva location, buccal capsule anatomy (integrated or formed by articulating plates), the length of spicule and gubernaculum, number of plates of buccal capsule, and presence or absence of vulvar papillae.

**Results:**

The hookworm species from Asian badger, here named as *Arthrostoma leucurus* sp. n., was different from the previously described ten *Arthrostoma* species. The phylogenetic tree based on the *cox*1 gene showed that *Arthrostoma leucurus* sp. n. formed a separate clade, as a sister group to *Ancylostoma* and *Uncinaria* species.

**Conclusion:**

*Arthrostoma leucurus* sp. n., the eleven validated *Arthrostoma* species, was identified from Asian badger in China.

**Supplementary Information:**

The online version contains supplementary material available at 10.1007/s11686-022-00587-5.

## Introduction

Hookworms (Nematoda: Ancylostomatidae) represent the most common soil-transmitted helminths, causing anemia and malnutrition in humans, domestic and wild animals [1–3]. The subfamilies, tribes, genera and even species of the superfamily Ancylostomatoidea are identified according to key characters, including anterior extremity bent dorsally or anterodorsally, oral opening with cutting plates or teeth, position of the vulva usually at the junction of posterior thirds of body or in the middle, buccal capsule integrated or formed by articulating plates, spicule length, gubernaculum length, number of plates in buccal capsule, presence or absence of vulvar papillae, host range and geographical distribution [4]. Within the subfamily Arthrocephalinae, the genera *Placoconus*, *Arthrocephalus* and *Arthrostoma* differ mainly in the number of articulated plates that form the buccal capsule, but also in parasitized hosts and their geographic distribution. Briefly, the genus *Placoconus* consists of hookworms with buccal capsule formed by five articulating plates, and host range including Procyonidae, Mustelidae, and Ursidae in North and South America. Buccal capsule formed by six articulating plates, and parasitized hosts belonging to Viverridae in Asia and Africa characterize *Arthrocephalus* species. In addition, hookworms with buccal capsule consisting of eight or ten articulating plates, and host range including Felidae, Viverridae, Mustelidae or Canidae in Asia or Pacific Oceania belong to *Arthrostoma*. To date, the genus *Arthrostoma* includes ten species (*Arthrostoma miyazakiense*, *A*. *felineum*, *A*. *vampira*, *A*. *spatulatum*, *A*. *longspiculum*/*longispicula*, *A*. *tunkanati*, *A*. *cheni*, *A*. *guilhoni*, *A*. *hunanensis,* and *A*. *brevispicula*). The first nine species were found in various families of carnivores (Felidae, Mustelidae, Canidae and Viverridae) [4–12]. The last one was found in *Manis pentadactyla allmanni* [5]. The information on species, hosts, geographic distribution and references are shown in Table 1.

**Table 1:** Data of mainly diagnostic characters of the 11 species of *Arthrostoma* Cameron, 1927 (Nematoda: Ancylostomatidae)

*Arthrostoma* species	Spicule length	Gubernaculum length	Number of plates in buccal capsule	Vulvar papillae	Host and country	Original references	Re-cited references
*Arthrostoma leucurus* sp. n.	0.602 ± 0.028 mm	71 ± 6 µm	10	Absent	*Meles leucurus*. China	In this study	/
*A. tunkanati*	Less than 0.5 mm	40–54 µm	10	Present	*Felis bengalensis horsfieldi.* Nepal	Nglis and Ogden, 1965	[4]
*A. vampira*	0.270–0.310 mm	60–75 µm	8	Present	*Mydaus marchei.* Philippines	[9]	/
*A. spatulatum*	0.352–0.387 mm	About 65 µm	8	Present	*Pardofelis marmorata marmorat*. Thailand	[6]	/
*A. guilhoni*	3.2 mm		8	Absent	*Arctictis binturong*. Vietnam	Le-Van Hoa, 1969	[4]
*A. cheni*	1.92–2.05 mm	135–154 µm	8	Present	*Paradoxurus minor exitus, Paguma larvata larvata.* China	Kou, 1958	[9]
*A. longespiculum/ A. longispicula*	1.19–2.19 mm	95–108 µm	8	Present	*Paguma larvata intrudens, Viverricula malaccensis, Paradoxurus philippensis, P. hermaphroditus. *Vietnam, China, India, Philippines, Thailand	(Sandground, 1933; Maplestone, 1931), [9]	[9, 10]
*A. miyazakiense*	1.100–1.400 mm	80–122 µm	10	Present	*Nyctereutes procyonoide viverrinus*. Japan	Sung-Shik Shin, 2007	[7, 12]
	1.440 mm	–				Nagayosi, 1955	
	1.230–1.400 mm	92–110 µm				Yoshida 1965	
*A. felineum*	About 0.9 mm	–	8	Present	F*elis bengalensis horsfieldi, F. temmincki, F. domesticu*. Sumatra, Nepal, Palestine	[8]	/
*A. hunanensis*	1.245–1.292 mm	82–91 µm	8	Present	*Felis bengalensis.* China	[11]	/
*A. brevispicula*	0.320–0.346 mm	60–70 µm	8	–	*Manis pentadoctyla allmanni*. China	Wang, 1984	[5]

In previous studies, *Uncinaria criniformis* and *Uncinaria stenocephala* were found in Eurasian badger (*Meles meles*) from Spain and the UK [13, 14]. In addition, *Ancylostoma taxideae* sp. n. was reported to occur in American badgers (*Taxidea taxus*) from the USA [15], *Ancylostoma braziliense*, *Tetragomphius melis,* and *Tetragomphius procyonis* in the Japanese badger (*Meles meles anakuma*) from Japan [16, 17], and *Tetragomphius arctonycis* sp. n. in the hog badger (*Arctonyx collaris*) from Japan [18]. Here, a novel hookworm species belonging to the genus *Arthrostoma* is described from Asian badger (*Meles leucurus*) in northwestern China.

## Materials and Methods

### Parasite Collection

In May 2018, fresh carcasses of two badgers found in Nilka County (Xinjiang Uygur Autonomous Region, northwestern China) were sent to our laboratory. After a routine necropsy of the small intestine, nineteen hookworms were found and further analyzed. This study was approved by the Animal Ethics Committee of Shihezi University (Approval No. A2018-143-01).

### Morphological Examination

Collected hookworms were examined with Olympus DP70 digital camera (Olympus, Tokyo, Japan) after treatment with 5–10% hot formalin [19]. The optical microscope was used to observe the microscopic structural characteristics of hookworms, such as articulating plates, buccal capsule plate arrangement, the length of spicule, gubernaculum and esophagus, bursa copulatrix, stylet, cutting plates, ventral lancets, submedian papillae, amphids, dorsal gland, nerve ring, valves on the opening of esophagus, genital cone, dorsal ray of the bursa, vulva, spine-like point of female tail and so on.

### Molecular Identification

DNA was extracted from hookworm adults using the Universal Genomic DNA Extraction Kit (TaKaRa, Dalian, China) according to the manufacturer's protocol instructions. The nearly full length (1209-bp-long part) of mitochondrial cytochrome *c* oxidase subunit 1 (*cox*1) gene was amplified by polymerase chain reaction (PCR) method. The PCR reactions were performed in a total volume of 25 µl containing 10 × Buffer, 200 mM of each dNTP, 1.5 mM MgCl_2_, 10 pmol of each primer and 2.5 U of platinum *Taq* DNA polymerase. 2 µl of genomic DNA was used as template. The *cox*1 gene was amplified using the forward primer 5′-TCT AAT CAT AAG GAT ATT GGA AC-3′ and reverse primer 5'-ACA GAA TAA ACA TCA GGA TAA TC-3′. The cycling conditions included an initial denaturation at 94 °C for 5 min, followed by 35 cycles at 94 °C for 30 s, 47 °C for 30 s, and 72 °C for 45 s, with a final extension at 72 °C for 8 min. Each PCR assay included a negative control (distilled water instead of hookworm DNA template). Amplified PCR products were electrophoretically separated in a 1% (*w*/*v*) agarose gel stained with GoldView II. The PCR products were purified using the TIANgel Midi Purification Kit (Tiangen, Beijing, China) and sequenced. Obtained sequences were edited and compared to GenBank data using BLAST program (http://www.ncbi.nlm.nih.gov/BLAST/). The sequences from this study were deposited in the GenBank database (accession numbers MW517831 and MW517832). Phylogenetic analyses, including *cox*1 sequences available in GenBank, were conducted by the maximum-likelihood (ML) method using 1000 bootstrap replicates by the MEGA 7.0 software.

## Results

**Fig. 1:**
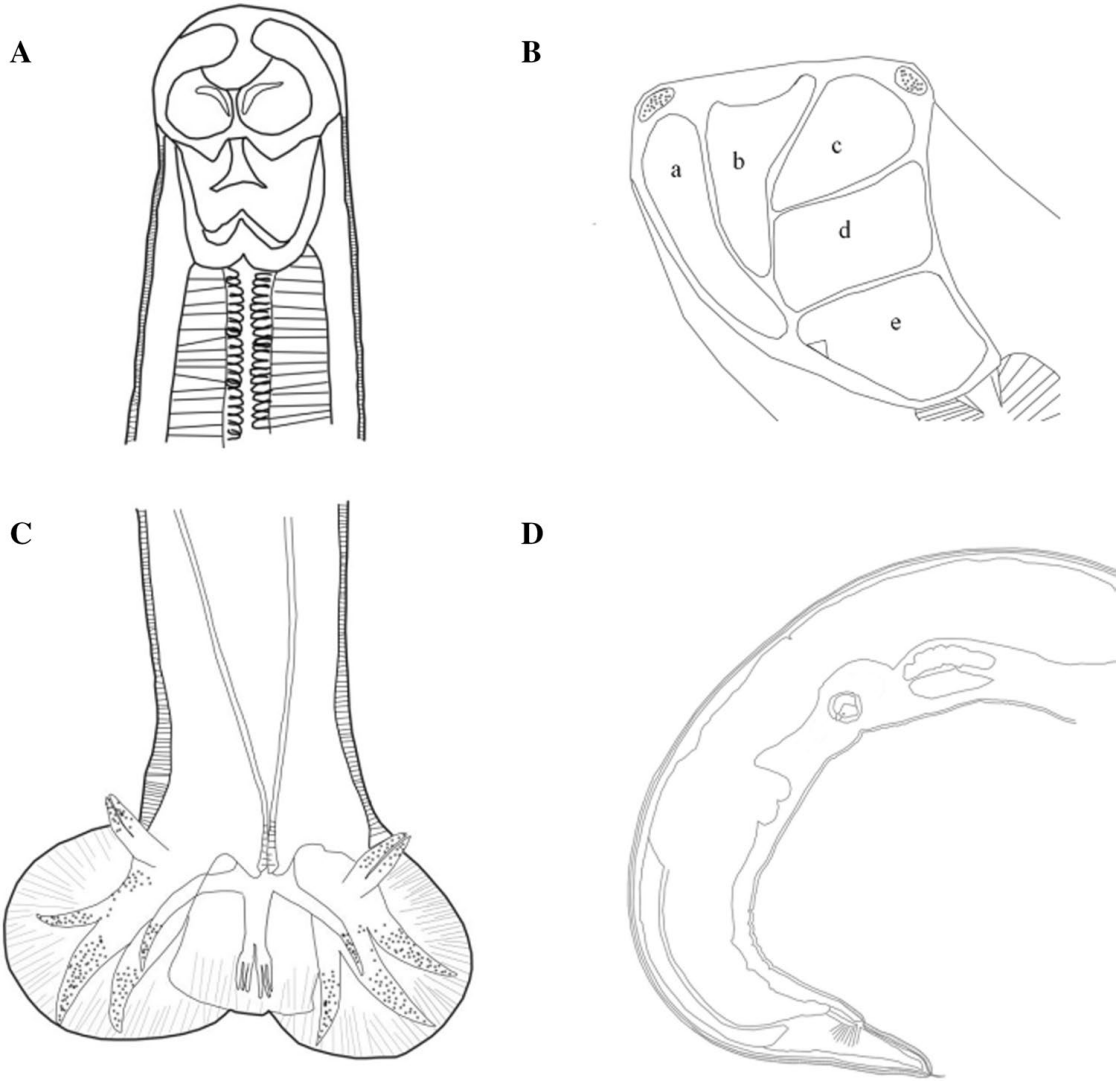
Drawings of *Arthrostoma leucura* sp. n. (Nematoda: Ancylostomatidae) from the Asian badger *Meles leucurus* Hodgson (Carnivora: Mustelidae) from Nilka County, Xinjiang Province, China. **A** Semilunar cutting plate; **B** buccal capsule, lateral view (a: ventral plates, b: ventrolateral plates, c: dorsolateral plates, d: lateral plates, e: basal plate); **C** male, bursa copulatrix; **D** female, vulvar papillae are absent

**Fig. 2:**
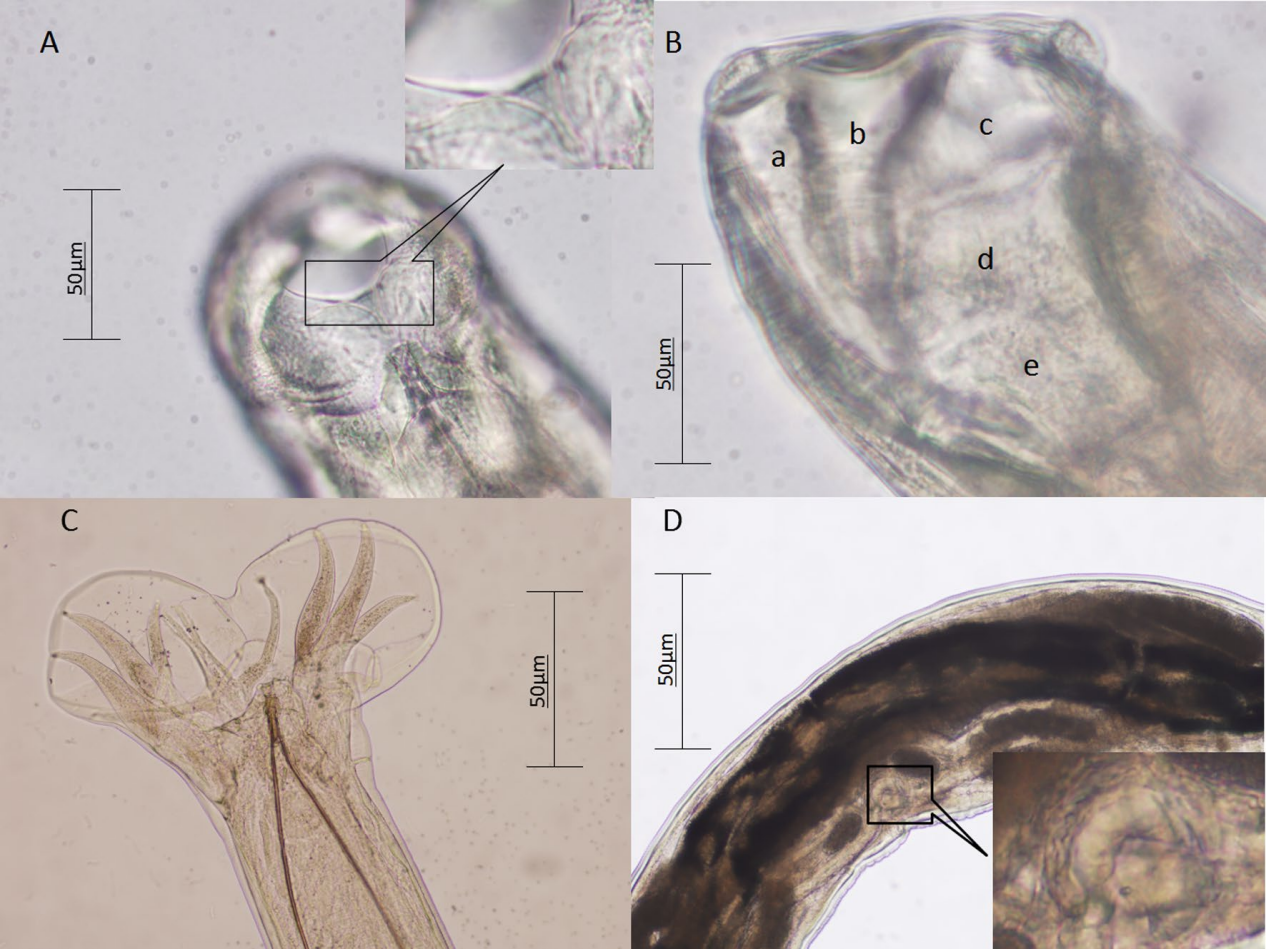
Light microscopic photographs of *Arthrostoma leucura* sp. n. (Nematoda: Ancylostomatidae) from the Asian badger *Meles leucurus* Hodgson (Carnivora: Mustelidae) from Nilka County, Xinjiang Province, China. **A** Semilunar cutting plate; **B** buccal capsule, lateral view (a: ventral plates, b: ventrolateral plates, c: dorsolateral plates, d: lateral plates, e: basal plate); **C** male; **D** female, vulvar papillae are absent

A total of 19 hookworm specimens (9 males and 10 females) were collected from the small intestines of two wild Asian badgers. The hookworms had the following morphological characteristics. Anterior extremity was bent dorsally. A stylet was located in the front of the buccal capsule. Two semilunar cutting plates were present on the margin of the buccal capsule. A small blunt ventral tooth was found near each side of the base of buccal capsule. The buccal capsule comprised 10 articulated plates including 3 ventral, 2 ventrolateral, 2 dorsolateral, 2 lateral and 1 basal plates. Four small submedian papillae and two amphids were located around the mouth. Two head glands were well-developed and located at the margin of the oral opening. Three lobed valves were present in the front of the opening of the esophagus. A nerve ring surrounded the mid-esophageal region. Based on morphological examination of three males and four females, the following measurements were obtained. *Male*: Body length was 6.18 ± 0.41 mm, maximum width at mid-body 249 ± 6 µm, buccal capsule width 65 ± 5 µm and length 100 ± 6 µm, esophagus length 574 ± 12 µm and width 116 ± 4 µm, the length of nerve-ring posterior to cephalic extremity 354 ± 17 µm, spicule length 602 ± 28 µm, gubernaculum length 71 ± 6 µm, genital cone at the posterior of the body, the wing membrane with transverse striations and two digitations on both sides of body terminus, and bursa with large, paired, ventrolateral lobes and small dorsal lobe with transverse striations. Dorsal ray was thick and bifurcated from anterior part into 2 branches, and each branch was further divided into 3 sub-branches. The arcuate externodorsal rays arise from the root of dorsal ray. Lateral rays were slender, tapering and arcuate with a common stem. Anterolateral rays bent anteriad, and medio- and posterolateral rays projected in parallel, extending to the edge of bursa. Antero- and posteroventral rays merged at base and then divided, continuously parallel, deepened into cleft. *Female*: Body length was 7.46 ± 1.06 mm, width 271 ± 28 µm. Buccal capsule width was 73 ± 13 µm, and length was 110 ± 2 µm. Esophagus length was 609 ± 28 µm and maximum width 121 ± 3 µm near the base. The length of nerve-ring posterior to cephalic extremity was 357 ± 7 µm. Excretory pore measured 158 ± 5 µm from its anterior to posterior end. Vulvar papillae were absent. The main morphological characteristics of hookworms are shown in Figs. 1 and 2.

These specimens belonged to the family Ancylostomatidae, subfamily Ancylostomatinae, which are haematophagous parasites of carnivores and omnivores. According to the standard identification procedure summarized in the book "Key to the nematode parasites of vertebrates" [4], the following steps were essential for the morphological identification of hookworms in this study. Firstly, because the anterior extremity was bent dorsally, the oral opening was armed with cutting plates, and the vulva was located at the junction of posterior thirds of body, the hookworms in this study belonged to Ancylostomatinae. Secondly, because the buccal capsule was formed by articulating plates, and cutting plates were poorly developed, all examined hookworms belonged to Arthrocephalinea. Thirdly, because the buccal capsule consisted of 10 articulating plates, the new hookworm species belonged to the genus *Arthrostoma* [4]. Fourthly, according to spicule length (0.602 ± 0.028 mm), gubernaculum length (71 ± 6 µm), number of buccal plates (10), absence of vulvar papillae, parasitized host (*Meles leucurus*) and geographical location (northwestern China), the hookworm from Asian badger, named here as *Arthrostoma leucurus* sp. n., was different from all previously described ten *Arthrostoma* species (shown in Table 1).

### Taxonomic Summary

- Type host: *Meles leucurus* Hodgson (Carnivora: Mustelidae).
- Type locality: Nilka County (N46° 40′ 40.63″, E90° 22′ 23.97″), Xinjiang Province, China.
- Site of infection: Small intestine.
- Prevalence and intensity of infection: 100% (2/2).
- Type specimens: Holotype male, allotype female and paratypes: 9 males, 10 females.
- Host specimens deposited: School of Medicine, Shihezi University.
- Etymology: The specific epithet is *Arthrostoma leucurus* sp. n.
- ZooBank.org: pub:AF934C3C-E2B0-43B7-AD75-2915A8A197CB.

### Nucleic Acid and Phylogenetic Analysis

**Fig. 3:**
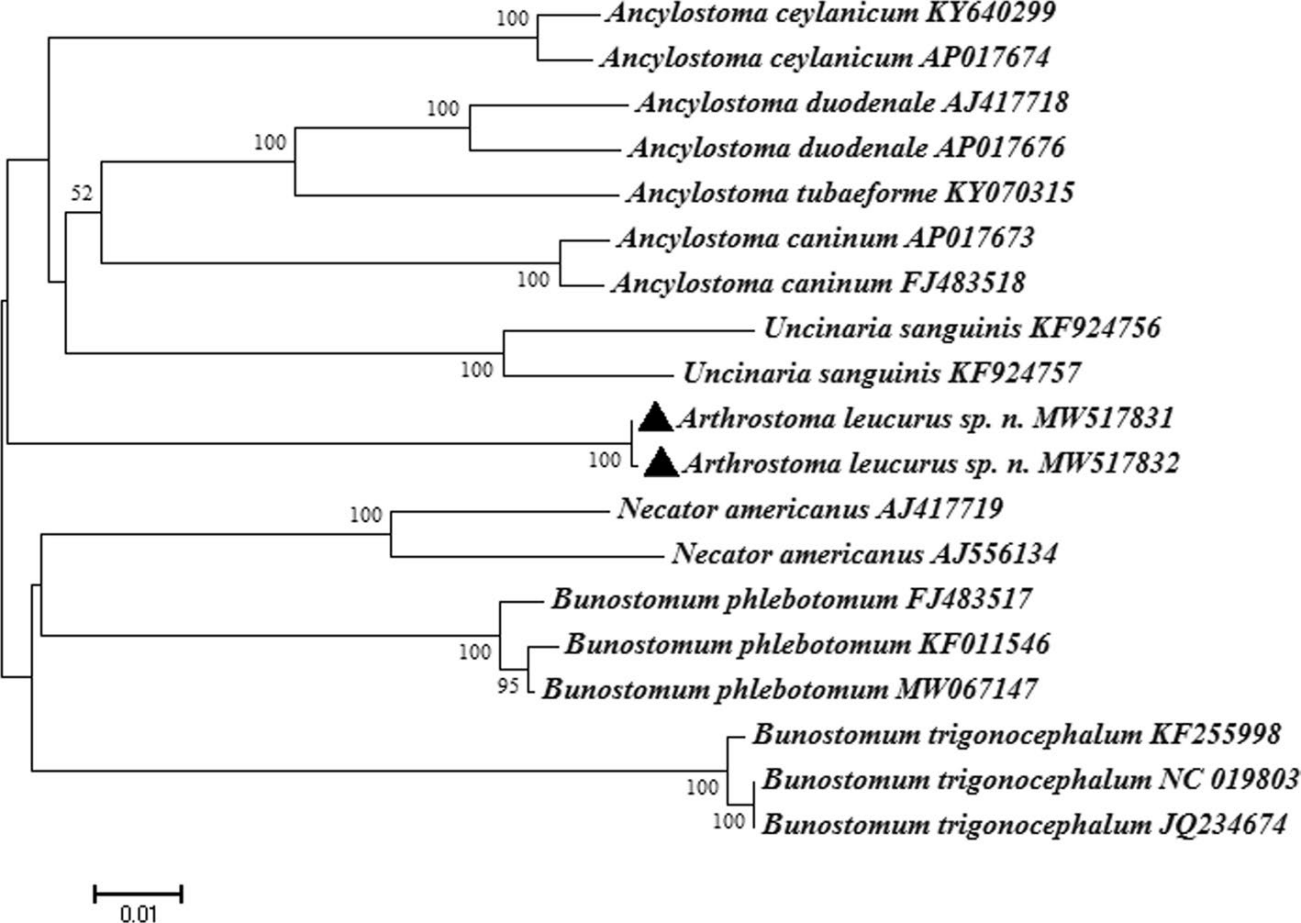
Phylogenetic relationships between *Arthrostoma leucurus* sp. n. and other taxa of the family Ancylostomatidae, as inferred from sequences of *cox*1 mtDNA (1209 bp). The tree was made by the Maximum Likelihood (ML) method. Sequence of *Bunostomum trigonocephalum* (GenBank accession No: KF255998, NC_019803, JQ234674) was included as an outgroup. The phylogenetic trees were generated by MEGA 7.0 from the aligned amino acid sequences of the *cox*1 gene of *Arthrostoma leucurus* sp. n. Sequences of *Arthrostoma leucuru*s sp. n. obtained in this study are indicated by solid triangles

The PCR amplification of the mtDNA from adult of *Arthrostoma leucurus* n. sp. yielded a product of 1209 bp. The topology of the *cox*1 phylogenetic tree was confirmed by high bootstrap supports (Fig. 3). In general, all members of Ancylostomatidae included in our analyses formed a monophyletic group. The isolates obtained in this study appeared on the tree closely related to the *Uncinaria sanguinis* (GenBank accession number KF924757, KF924756) from Australian sea lions (*Neophoca cinerea*) in Australia. BLAST analysis based on mitochondrial *cox*1 gene indicated that *Arthrostoma leucurus* sp. n. was related closest to members of genus *Uncinaria*, i.e., in a 360-bp-long sequence it had the following rates of identity: 87.91% with the sequences of *Uncinaria lucasi* (GenBank accession number MT154431), 87.25% with *U*. *lyonsi* (accession number MT154507), 89.54% with *Uncinaria* sp. (accession number KY465459), 88.24% with *U*. *sanguinis* (accession number KF924756), and in a 441-bp-long sequence the rate of identity was 88.01% with *U*. *stenocephala* (accession number MT361502). The similarities between sequences of *Arthrostoma leucurus* sp. n. and the five *Uncinaria* species available in GenBank were in the range of 89.54–87.25% and 97.95–98.52% according to nucleotides and amino acids, respectively (Supplementary Fig. 1). Phylogenetic analysis showed that the two sequences of *Arthrostoma leucurus* sp. n. belonged to a separate clade, as a sister group to *Ancylostoma* and *Uncinaria* species (Fig. 3).

## Discussion

According to the classical taxonomy of Ancylostomatidae, it includes two subfamilies: Ancylostomatinae and Bunostominae [4]. Hosts of the former group are carnivores and omnivores, while hosts of the latter are omnivores and herbivores [4]. Regarding molecular results, although prior to this study *cox*1 sequence data were not available from the genus *Arthrostoma* in GenBank (limiting the conclusions that can be drawn here), results of the phylogenetic analyses performed here are still informative and interesting (Fig. 3). These are consistent with the host range and the structure on the margin of the oral opening, i.e., (i) the genera of *Necator*, *Bunostomum*, *Uncinaria* and *Arthrostoma* have omnivorous and herbivorous hosts and possess cutting plates, whereas (ii) species of *Ancylostoma* genus have hosts from orders Carnivora, Primates, Edentata, Rodentia and Suidae, and possess teeth rather than cutting plates. The hosts of *Arthrostoma* genus include Felidae, Viverridae, Canidae, Ursidae and Mustelidae [4]. According to the parasitized host and the morphological characteristics of *Arthrostoma leucurus* sp. n., it is different from all ten known species of *Arthrostoma* [4–12].

In recent years, human activities and overhunting have resulted in a dramatic decline in the population size of Asian badgers [20]. In addition, some pathogens, such as *Sarcocystis lutrae*, *Babesia* sp. Meles-Hu1, *Toxoplasma gondii* and *Brucella melitensis* [21–24], could infect Asian badgers, thus contributing to decrease in Asian badger populations. In the present study, a large number of mature and young erythrocytes were found in the intestine of hookworms (Supplementary Fig. 2). This indicates that the novel hookworm can cause blood loss in Asian badgers. Therefore, in the context of this host species, further aspects of hookworm infection should be investigated in the near future, including the taxonomic diversity of the causative agents, their epidemiology and pathophysiological effects.

## Supplementary Information

Below is the link to the electronic supplementary material.

Supplementary file1 (TIF 6965 kb)

Supplementary file2 (TIF 3793 kb)
