# Supplementary figures and images for: Arthrostoma leucurus sp. n. (Nematoda: Ancylostomatidae), A New Hookworm Species Isolated from Asian Badger in China
Source: Acta Parasitol. 2022 Jul 23;67(3):1447–54. doi: 10.1007/s11686-022-00587-5 (PMC9399214; doi:10.1007/s11686-022-00587-5)

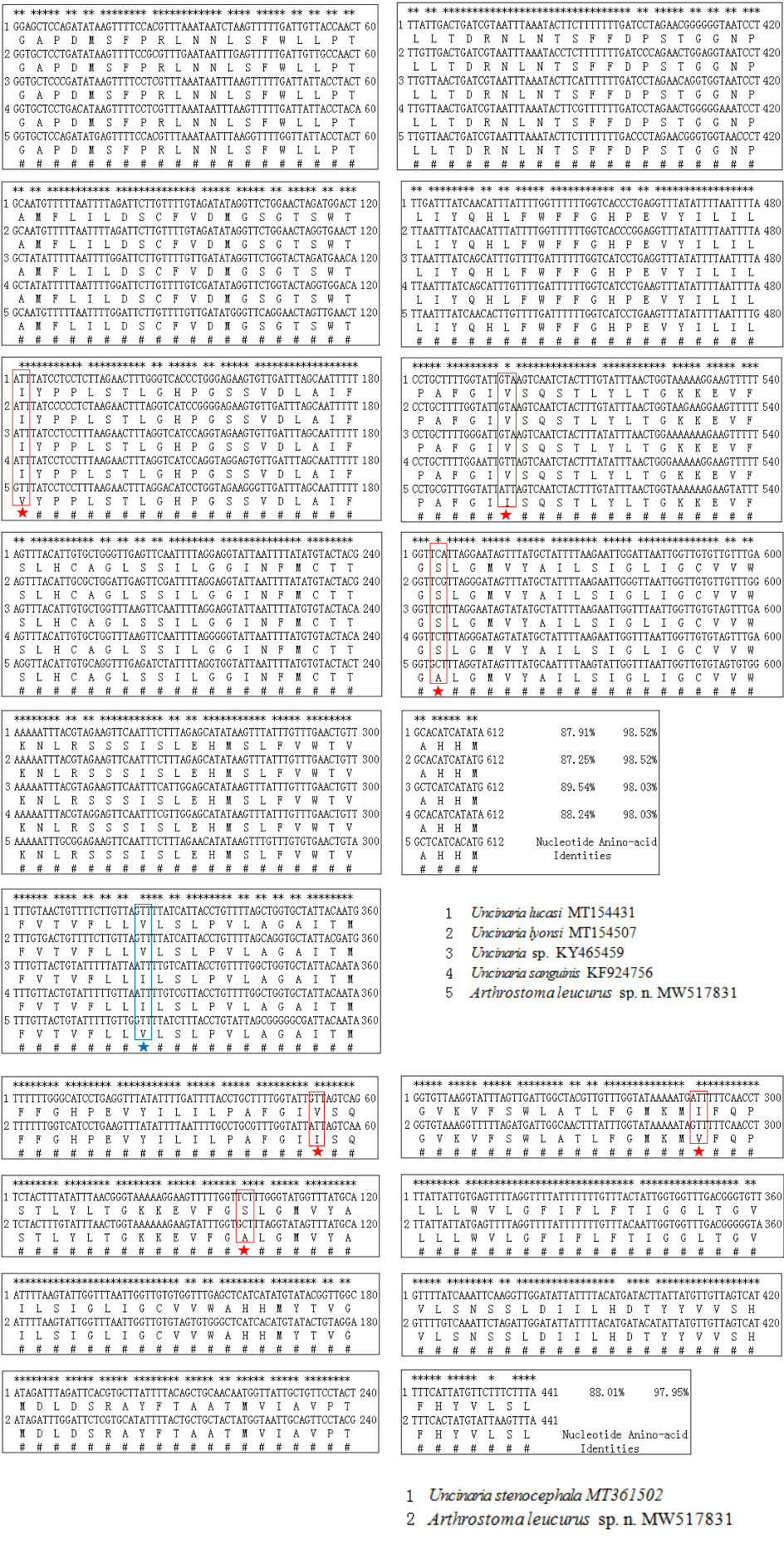

Supplement: Supplementary file 1 — Supplementary file1 (TIF 6965 kb) [file 11686_2022_587_MOESM1_ESM.tif]

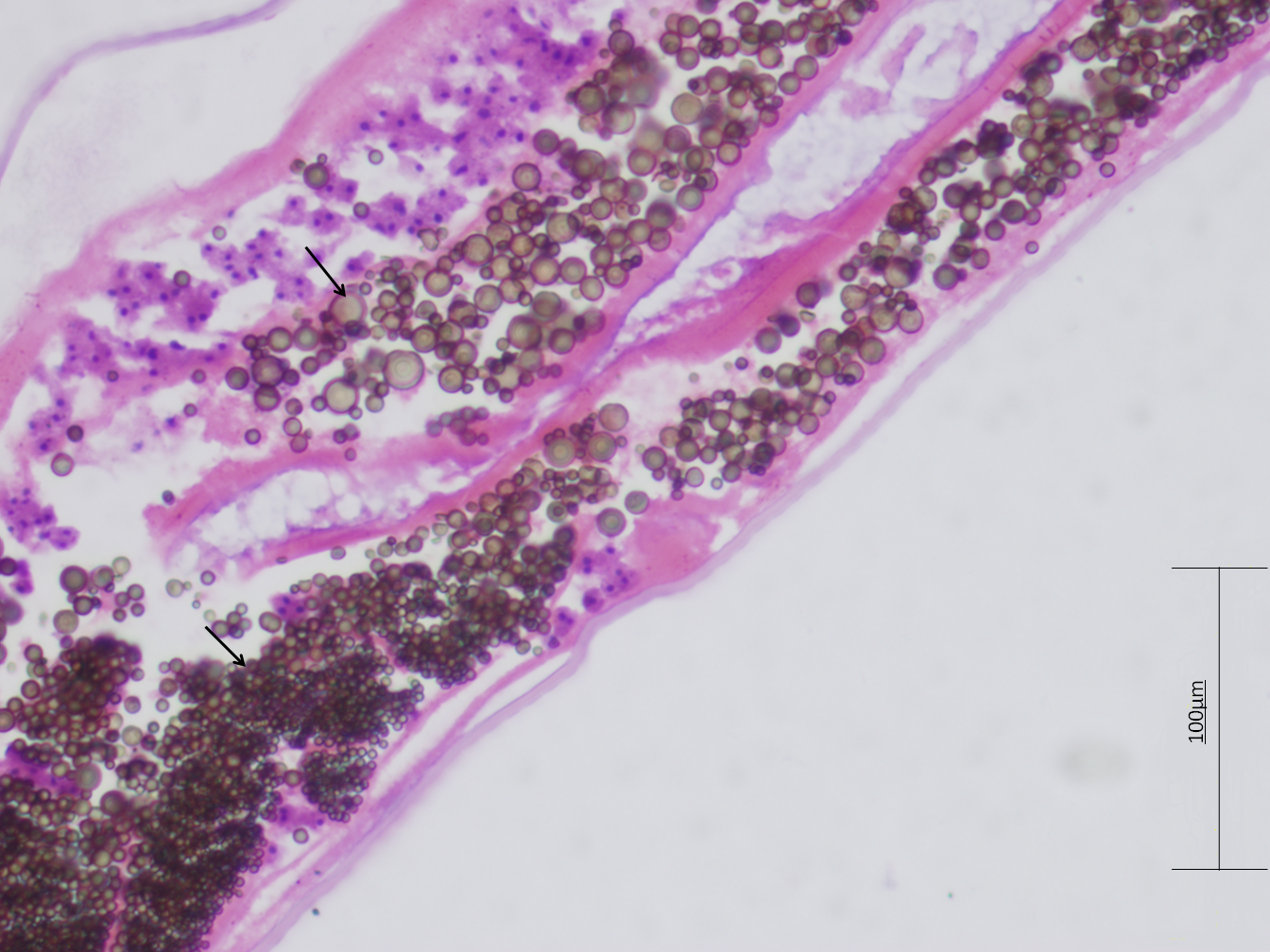

Supplement: Supplementary file 2 — Supplementary file2 (TIF 3793 kb) [file 11686_2022_587_MOESM2_ESM.tif]
